# Secondary infections and long-term outcomes among hospitalized elderly and non-elderly patients with severe acute respiratory syndrome coronavirus 2 (SARS-CoV-2) and treated with baricitinib: a comparative study from the national centre of Hungary

**DOI:** 10.1007/s11357-024-01099-y

**Published:** 2024-02-17

**Authors:** Zsófia Gáspár, Bálint Gergely Szabó, Hajnalka Andrikovics, Andrea Ceglédi, Martin RAJMON, Anita Ábrahám, Zsuzsanna Várnai, Noémi Kiss-Dala, János Szlávik, János Sinkó, István Vályi-Nagy, Botond Lakatos

**Affiliations:** 1 National Institute of Haematology and Infectious Diseases, Central Hospital of Southern Pest, Albert Flórián Street 5-7., 1097 Budapest, Hungary; 2 School of PhD Studies, Semmelweis University, Üllői Street 26., 1085 Budapest, Hungary; 3 Departmental Group of Infectious Diseases, Department of Internal Medicine and Haematology, Semmelweis University, Üllői Street 26., 1085 Budapest, Hungary; 4 Laboratory of Molecular Genetics, National Institute of Haematology and Infectious Diseases, Central Hospital of Southern Pest, Albert Flórián Street 5-7., 1097 Budapest, Hungary; 5 Faculty of Medicine, Semmelweis University, Üllői Street 26., 1085 Budapest, Hungary; 6 Department of Transfusion Medicine, Semmelweis University, Üllői Street 26., 1085 Budapest, Hungary

**Keywords:** Coronavirus disease 2019, Severe acute respiratory syndrome coronavirus 2, COVID-19, SARS-CoV-2, Immunomodulation, Baricitinib, Elderly, Secondary infection

## Abstract

Baricitinib is considered a first-line treatment for severe acute respiratory syndrome coronavirus 2 (SARS-CoV-2)-infected adult patients with an associated cytokine storm syndrome (CSS). Our objective was to compare rates of secondary infections and long-term outcomes of elderly and non-elderly patients who received baricitinib for COVID-19. We conducted a single-centre observational study between November 2020 and September 2023, focusing on hospitalized adult SARS-CoV-2 patients with CSS, categorized as elderly (≥ 65 years) and non-elderly (< 65 years). Enrolment, severity stratification, and diagnosis of infectious complications followed predefined criteria. Outcomes of all-cause mortality and rates of non-severe and severe secondary infections were assessed at 1-year post-treatment initiation. Kaplan–Meier analysis was performed for survival analysis. In total, 490 patients were enrolled (median age 65 ± 23 (21–100) years (years, median ± IQR, min–max); 49.18% elderly; 59.59% male). Elderly patients were admitted to the hospital significantly earlier (7 ± 5 days vs. 8 ± 4 days; *p* = 0.02), experienced a higher occurrence of severe COVID-19 (121/241, 50.21% vs. 98/249, 39.36%; *p* = 0.02), and required the use of non-invasive ventilation at baseline (167/225, 74.22% vs. 153/236, 64.83%; *p* = 0.03). At 1 year, all-cause mortality was significantly higher in the elderly subgroup (111/241, 46.06% vs. 29/249, 11.65%; *p* < 0.01). At 90 days and 1 year, rates of any severe secondary infection were also more prevalent among the elderly (56/241, 23.24% vs. 37/249 14.86%; *p* = 0.02 and 58/241, 24.07% vs. 39/249, 15.66%; *p* = 0.02). In conclusion, elderly SARS-CoV-2-infected patients experience a more severe clinical course, higher secondary infection rates, and increased risk for long-term mortality, regardless of immunomodulatory therapy.

## Introduction

For severe acute respiratory syndrome coronavirus 2 (SARS-CoV-2)-infected adult patients who exhibit a severe clinical course at baseline and complicated by cytokine storm syndrome (CSS), immunomodulatory therapy is recommended in conjunction with remdesivir and corticosteroids [1, 2]. Both the Infectious Diseases Society of America (IDSA) and the European Society of Clinical Microbiology and Infectious Diseases (ESCMID) Coronavirus disease 2019 (COVID-19) guidelines recommend the use of baricitinib, an immunomodulatory drug that has been shown to lower mortality among patients with severe COVID-19, requiring high-flow oxygen or non-invasive ventilation [1, 2]. Initial data suggested that secondary bacterial and fungal opportunistic infections could be potential side effects of baricitinib administration, possibly leading to prolonged hospitalization and increased mortality rates [3, 4]. Consequently, some national guidelines, such as the one from Hungary, advised prophylactic antimicrobial measures, e.g., the suggestion of a 3- to 6-month regimen of co-trimoxazole and acyclovir after the completion of baricitinib therapy [5]. However, recent clinical trials do not indicate significant differences in secondary infection rates between patients receiving the standard-of-care (SOC) treatment alone and those receiving SOC with baricitinib [6, 7].

Age stands as a pre-defined risk factor for COVID-19 progression [8]. Moreover, in older patient populations, increased rates of secondary or nosocomial infections, prolonged hospitalization, and elevated mortality rates are probably anticipated, irrespective of COVID-19 [9]. Nevertheless, only a few studies addressed the disparities in clinical courses and outcomes of elderly versus non-elderly patients treated with baricitinib because of SARS-CoV-2 infection. Therefore, our objective was to assess the risk of secondary infections and long-term outcomes in elderly adults hospitalized with COVID-19 and receiving baricitinib, in contrast to non-elderly patients.

## Methods

### Study design and settings

A single-centre comparative observational study was conducted among adult patients infected with SARS-CoV-2 who received baricitinib as immunomodulatory therapy between November 28, 2020, and September 1, 2023, at the Central Hospital of Southern Pest, National Institute of Haematology and Infectious Diseases (Budapest, Hungary). Our centre serves as a national centre that experiences a substantial inflow of COVID-19 patients and possesses ≥ 150 beds to accommodate these cases. Treatment of COVID-19 patients was conducted in the framework of the CONTRAST (*COmparing Novel TReatment Strategies Against SARS-CoV-Two*) clinical trial, initiated by our centre. The trial was approved by the Scientific and Research Ethics Committee of the Hungarian National Medical Scientific Council (ETT-TUKEB IV/3937–1/2020/EKU). The National Institute of Pharmacy and Nutrition authorized the off-label use of baricitinib treatment. The research adhered to the principles of the Helsinki Declaration and national ethical standards. Each enrolled individual provided written informed consent. Data reporting followed the guidelines outlined in the STROBE (*Strengthening the Reporting of Observational Studies in Epidemiology*) Statement.

### Patient eligibility and inclusion

All admitted adult patients with respiratory real-time polymerase chain reaction (RT-PCR) confirmed SARS-CoV-2 infection were considered for inclusion. Attending physicians conducted daily patient screenings for inclusion, following predefined inclusion and exclusion criteria. Inclusion criteria comprised (1) age of ≥ 18 years, (2) severe clinical course as per the criteria established by the World Health Organization (WHO) [8], (3) COVID-19-associated CSS based on clinical course and laboratory parameters elaborated below [10], and (4) reception of SOC and baricitinib therapy (as defined below). Tocilizumab became accessible in April 2020 and demonstrated efficacy in managing COVID-19-associated CSS. In contrast, baricitinib received emergency use authorization in November 2020 for off-label usage in the context of COVID-19-associated CSS, subsequently largely supplanting tocilizumab. Consequently, owing to ethical considerations, there existed a brief overlap period during which some patients received both tocilizumab and baricitinib. These individuals were also incorporated into the study cohort. The exclusion criteria were (1) the unavailability of patient data via the hospital electronic database during follow-up and (2) patients with a severe clinical course and a suspected CSS receiving only tocilizumab. For statistical analysis, the allocation of patients into elderly and non-elderly subgroups was performed, defined as ≥ 65 years and < 65 years, respectively.

### Diagnostic and therapeutic strategies

Each patient referred to our centre underwent baseline evaluation by an attending physician to determine the necessity of hospitalization. For assessment, SARS-CoV-2 diagnosis confirmation relied on the oro- or nasopharyngeal sample RT-PCR positivity following the European Centre for Disease Prevention and Control COVID-19 diagnostic definition [11]. COVID-19 severity and risk factors for disease progression were evaluated in accordance with WHO criteria [8]. Classification of individuals as fully vaccinated against COVID-19 was established ≥ 14 days after the completion of the recommended dosing schedule of a nationally authorized vaccine (Janssen, Moderna, Oxford-AstraZeneca, Pfizer-BioNTech, Sinopharm, and Sputnik V). Partial vaccination status was attributed to individuals who had solely received one dose of any of the aforementioned vaccines (with the exception of the Janssen vaccine). At baseline, laboratory tests including blood absolute white blood cell, neutrophil granulocyte, lymphocyte, platelet counts, serum c-reactive protein (CRP), plasma interleukin-6, serum ferritin, serum lactate dehydrogenase (LDH), and plasma D-dimer were obtained, alongside a chest computed tomography (CT) scan. Chest CT scan findings were assessed by attending radiologists, who based their evaluation on the lung involvement of COVID-19 pneumonitis and secondary pneumonia, relative to the extent of normal lung tissue. An empirical estimated percentage, ranging from 0 to 100, was assigned, and this was referred to as the CT infiltration rate.

COVID-19-associated CSS was defined by ≥ 1 clinical and ≥ 2 biochemical criteria [10]. Clinical criteria encompassed (1) persistent fever (≥ 38 °C for ≥ 3 days), (2) resting oxygen saturation of ≤ 94% or an arterial partial O_2_ tension/inspirational O_2_ fraction of ≤ 300 mmHg with or without tachypnoea or dyspnoea on room air, and (3) indicators of multi-organ failure (acute respiratory distress syndrome, circulatory shock, acute hepatic failure, acute renal failure, acute pancytopenia, acute coagulopathy, and acute delirium) [10]. ARDS was defined according to the Berlin criteria [12]. Biochemical criteria included (1) serum IL-6 three times above the upper limit of normal (ULN); (2) serum ferritin ≥ 600 µg/l, (3) serum LDH above the ULN, (4) plasma D-dimer ≥ 1000 ng/l, and (5) serum CRP ≥ 75 mg/dl.

Patient evaluation took place on a daily basis. Laboratory tests and arterial blood gases were collected every other day. Chest CT scans were conducted in cases of deteriorating clinical conditions. New-onset fever or clinical instability prompted the acquisition of blood cultures. Microbiological diagnostics were performed at the Core Microbiology Laboratory of Central Hospital of Southern Pest.

The standard-of-care (SOC) for the COVID-19 treatment of hospitalized patients exhibiting a severe clinical course comprised of remdesivir (and before remdesivir distribution, favipiravir, or hydroxychloroquine), dexamethasone, and supportive therapies (low-molecular-weight heparin, oxygen supplementation, respiratory support, intravenous fluids, antipyretics, expectorants, and bronchodilators). If CSS was suspected, the administration of an immunomodulatory drug was promptly initiated. Starting from April 2020, tocilizumab became accessible in Hungary; however, it was contraindicated for patients with significantly elevated serum aspartate aminotransferase and/or serum alanine aminotransferase levels, exceeding five times the ULN. Subsequently, as of November 2020, baricitinib also became available, although it was contraindicated in patients with swallowing difficulties. A daily administration of oral 4 mg baricitinib was maintained for at least 7 days. Initially, the treatment duration was set at 21 days [5]. However, in light of emerging evidence, the recommended maximum treatment duration has been defined as 14 days or until hospital discharge, as deemed necessary [1]. Clinical improvement carried more weight in the treatment duration decision than any specific patient characteristics. COVID-19 and secondary infection treatment allocations were in line with in-house and national guideline protocols [1, 2, 5].

### Data collection

During hospitalization, the following data were gathered from electronic medical records and clinical charts: (1) demographic information, (2) comorbidities and patient-level risk factors for disease progression, (3) COVID-19 vaccination status, (4) baseline data at COVID-19 diagnosis (symptom onset and severity, SARS-CoV-2 RT-PCR results, oxygen supplementation requirements, chest CT scan findings, and laboratory results), (5) clinical course of the disease (intensive care unit (ICU) admission, hospital length of stay (LOS), ICU LOS), (6) treatment strategies (initiation and duration of baricitinib treatment and additional treatments), (7) secondary infections (type of infection, collected specimen, causative organism, and date of secondary infection), and (8) clinical outcomes.

Following discharge from the hospital, patients underwent scheduled in-person follow-up examinations. If their clinical condition deteriorated or they developed new symptoms, unscheduled in-patient or telemedicine visits were also possible. The follow-up period extended up to 1 year after the completion of baricitinib treatment. After the follow-up ended, the patient status was re-evaluated through the National e-Health Infrastructure, and instances of secondary severe and non-severe infections were also documented.

### Outcomes

The primary outcome was all-cause mortality. All-cause mortality was defined as death attributed to any cause, regardless of COVID-19. Secondary endpoints were diagnosis of severe and non-severe secondary infections. A severe secondary infection was characterized as (1) an infection necessitating hospitalization for treatment or (2) manifesting during hospital stay and (3) demanding at least one dose of intravenous empirical antimicrobial drug. Non-severe secondary infection was defined as (1) an infection not requiring hospital admission for treatment, (2) not emerging during a hospital stay, and (3) not requiring intravenous empirical antimicrobial drug therapy. All outcomes were assessed at 30 days, 90 days, and 1 year from the initiation of baricitinib treatment.

### Statistical analysis

Categorical variables are presented as absolute numbers (*n*), along with their corresponding relative percentages (%). Continuous variables are described using the median with interquartile range (IQR) and the minimum–maximum range. The incidence rate is expressed as the number of cases per person-time, cumulated for 1000 persons over 1 year. The incidence rate ratio is presented along with its 95% confidence interval (CI). Statistical comparisons were conducted using Fisher’s exact test and the Mann–Whitney *U*-test. A two-tailed *p*-value of < 0.05 was considered statistically significant. For the primary outcome, a Kaplan–Meier survival analysis was performed along with log-rank testing. Kaplan–Meier curves were plotted using MedCalc (version 22.009). For the incidence rate analysis, STATA was used (version StataBE 18).

## Results

### Baseline characteristics

**Fig. 1:**
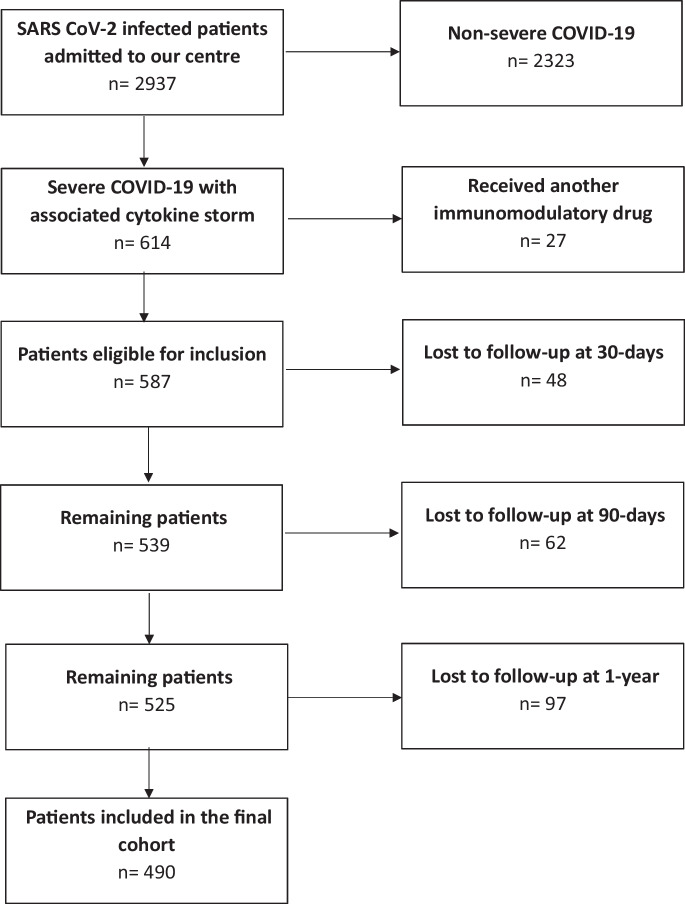
Patient inclusion flow chart

The patient flow chart is outlined in Fig. 1*.* A total of 490 patients were eligible for inclusion in the study. Details regarding baseline characteristics are provided in Table 1. The median age was 65 ± 23 (21–100) years, with 241/490 (49.18%) falling into the elderly subgroup. Overall, 292/490 (59.59%) were male. Among elderly patients, the prevalence of essential hypertension (185/241, 76.76% vs. 109/249, 43.78%; *p* < 0.01), chronic cardiovascular disease (106/241, 43.98% vs. 21/249, 8.43%; *p* < 0.01), chronic renal disease (41/241, 17.01% vs. 8/249, 3.21%; *p* < 0.01), chronic cerebrovascular disease (15/241, 6.22% vs. 5/249, 2.01%; *p* = 0.02), diabetes mellitus (88/241, 36.51% vs. 52/249, 20.88%; *p* < 0.01), and active oncological malignancy (22/241, 9.13% vs. 6/249, 2.41%; *p* < 0.01) were significantly higher. Chronic pulmonary disease (40/241, 16.60% vs. 28/249, 11.24%; *p* = 0.09), active haematological malignancies (19/241, 7.88% vs. 10/249, 4.02%; *p* = 0.08), systematic autoimmune disease (16/241, 6.64% vs. 11/249, 4.42%; *p* = 0.33), chronic systemic corticosteroid treatment (9/241, 3.73% vs. 7/249, 2.81%; *p* = 0.62), chronic immunosuppressive drug use (11/241, 4.56% vs. 11/249, 4.42%; *p* = 1.00), chronic alcohol use (16/241, 6.64% vs. 12/249, 4.82%; *p* = 0.44), tobacco use (25/241, 10.37% vs. 30/249, 12.05%; *p* = 0.57), and obesity (99/241, 41.08% vs. 117/249, 46.99%; *p* = 0.20) were equally distributed among subgroups. Male gender was more frequent among the non-elderly subgroup (121/241, 50.21% vs. 171/249, 68.67%; *p* < 0.01). The vaccination program started in Hungary in December 2020. However, 76.33% (*n* = 374) of patients were not vaccinated at the time of inclusion. Elderly patients were initially prioritized for vaccination, followed by non-elderly patients. Full vaccination status was tendentiously more frequent among the elderly (42/241, 17.43% vs. 29/249, 11.65%; *p* = 0.07).

**Table 1:** Baseline demographic and clinical characteristics of elderly and non-elderly patients treated with baricitinib for COVID-19

Parameter	Total (*n* = 490)	Elderly (*n* = 241)	Non-elderly (*n* = 249)	*p*-value
Age (years, median ± IQR, min–max)	65 ± 23 (21**–**100)	76 ± 13 (65**–**100)	53 ± 15 (21**–**65)	< 0.01
Male gender (*n*, %)	292 (59.59)	121 (50.21)	171 (68.67)	< 0.01
Comorbidities (*n*, %)				
Essential hypertension (*n*, %)	294 (60.00)	185 (76.76)	109 (43.78)	< 0.01
Chronic cardiovascular disease (*n*, %)	127 (25.92)	106 (43.98)	21 (8.43)	< 0.01
Chronic pulmonary disease (*n*, %)	68 (13.88)	40 (16.60)	28 (11.24)	0.09
Chronic renal disease (*n*, %)	49 (10.00)	41 (17.01)	8 (3.21)	< 0.01
Chronic hepatic disease (*n*, %)	36 (7.35)	22 (9.13)	14 (5.62)	0.17
Chronic cerebral disease (*n*, %)	20 (4.08)	15 (6.22)	5 (2.01)	0.02
Diabetes mellitus (*n*, %)	140 (28.57)	88 (36.51)	52 (20.88)	< 0.01
Active oncological malignancy (*n*, %)	28 (5.71)	22 (9.13)	6 (2.41)	< 0.01
Active hematologic malignancy (*n*, %)	29 (5.92)	19 (7.88)	10 (4.02)	0.08
Systemic autoimmune disease (*n*, %)	27 (5.51)	16 (6.64)	11 (4.42)	0.33
Chronic systemic corticosteroid treatment (*n*, %)	16 (3.27)	9 (3.73)	7 (2.81)	0.62
Chronic immunosuppressive drug use (*n*, %)	22 (4.49)	11 (4.56)	11 (4.42)	1.00
Chronic alcohol use (*n*, %)	28 (5.71)	16 (6.64)	12 (4.82)	0.44
Chronic tobacco use (*n*, %)	55 (11.22)	25 (10.37)	30 (12.05)	0.57
Obesity (*n*, %)	216 (44.08)	99 (41.08)	117 (46.99)	0.20
COVID-19 vaccination status (*n*, %)				
Non-vaccinated	374 (76.33)	176 (73.03)	198 (79.52)	0.11
Partially vaccinated	38 (7.76)	20 (8.30)	18 (7.23)	0.74
Fully vaccinated	71 (14.49)	42 (17.43)	29 (11.65)	0.07
No data	7 (1.43)	3 (1.24)	4 (1.61)	1.00
COVID-19 severity at baseline (*n*, %)				
No symptoms	11 (2.25)	8 (3.32)	3 (1.20)	0.14
Mild symptoms	166 (33.88)	66 (27.39)	100 (40.16)	< 0.01
Severe symptoms	219 (44.69)	121 (50.21)	98 (39.36)	0.02
Critical symptoms	68 (13.88)	32 (13.28)	36 (14.46)	0.79
Requirement of any oxygen support (*n*, %)	461 (94.08)	225 (93.36)	236 (94.78)	0.57
Oxygen support started at baseline (*n*, %)				
Low-flow nasal cannula	134/461 (29.07)	52/225 (23.11)	82/236 (34.75)	< 0.01
Venturi mask or non-invasive mechanical ventilation	320/461 (69.41)	167/225 (74.22)	153/236 (64.83)	0.03
Invasive mechanical ventilation	8/461 (1.74)	5/225 (2.22)	3/236 (1.21)	0.49
COVID-19 infiltrate expansion on chest CT scan at baseline (%, median ± IQR, min–max)	50 ± 40 (0**–**90)	50 ± 37.50 (0**–**90)	50 ± 40 (0**–**90)	0.03
Laboratory characteristics at baseline (median ± IQR, min–max)				
Blood absolute white blood cell count (× 10^9^/l)	6.73 ± 4.36 (2.04**–**245)	6.85 ± 4.73 (2.04**–**245)	6.57 ± 4.05 (2.44**–**96.39)	0.21
Blood absolute neutrophil granulocyte count (× 10^9^/l)	5.27 ± 3.71 (0.66**–**20.25)	5.42 ± 3.83 (1.27**–**20.25)	5.13 ± 3.71 (0.66**–**431)	0.48
Blood absolute lymphocyte count (× 10^9^/l)	0.88 ± 0.57 (0.06**–**232)	0.82 ± 0.51 (0.06**–**232)	0.95 ± 0.59 (0.21**–**137)	0.03
Blood absolute platelet count (× 10^9^/l)	196 ± 97 (13.40**–**682)	198 ± 102 (13**–**548)	196 ± 98 (21.00**–**682)	0.31
Serum CRP (mg/dL)	138 ± 122 (0.40**–**379)	140 ± 121 (0.70**–**365)	134 ± 121 (0.40**–**379)	< 0.01
Plasma interleukin-6 (pg/ml)	50.90 ± 69.30 (2.70**–**8350)	60.10 ± 96.73 (2.70**–**8350)	46.50 ± 56.00 (2.70**–**666)	< 0.01
Serum ferritin (ng/mL)	1054 ± 1203 (11.40**–**20262)	1020 ± 1243 (27.90**–**20262)	1103 ± 1137 (11.40**–**8750)	0.77
Serum LDH (IU/l)	789 ± 420 (53.00**–**2716)	749 ± 356 (53.00**–**2716)	845 ± 443 (264**–**2559)	< 0.01
Plasma D-dimer (ng/ml)	1073 ± 1059 (14.95**–**86220)	1276 ± 1296 (203**–**80208)	933 ± 755 (14.95**–**86220)	0.99
Remdesivir started at baseline (*n*, %)	476 (97.14)	233 (96.68)	243 (97.59)	0.60
Dexamethasone started at baseline (*n*, %)	490 (100)	241 (100)	249 (100)	n.a
Intensive care unit admission during hospitalization (*n*, %)	163 (33.27)	85 (35.26)	78 (31.33)	0.39
Time analysis (days, median ± interquartile range, min–max)				
Time from symptom onset to hospitalization	7 ± 4 (0**–**60)	7 ± 5 (0**–**60)	8 ± 4 (1**–**21)	0.02
Time from symptom onset to baricitinib treatment	9 ± 5 (0**–**69)	9 ± 5 (0**–**69)	9 ± 5 (2**–**62)	0.23
Time from COVID-19 diagnosis to baricitinib treatment	3 ± 7 (0**–**62)	2 ± 7 (0**–**35)	4 ± 6 (0**–**62)	0.07
Time from admission to first infectious complication	18 ± 32 (1**–**365)	19 ± 22.25 (1**–**362)	15 ± 79 (1**–**366)	0.46
Time from baricitinib treatment to first infectious complication	15 ± 31 (0**–**364)	15 ± 22 (1**–**362)	13 ± 80 (0**–**364)	0.32
Time from COVID-19 diagnosis to intensive unit care admission	1 ± 2 (0**–**23)	1 ± 2.25 (0**–**23)	1 ± 2 (0**–**14)	0.13
Length of hospital stay	15 ± 14 (2**–**213)	18 ± 16 (2**–**213)	14 ± 9 (2**–**188)	< 0.01
ICU length of hospital stay	15 ± 14 (2**–**213)	18 ± 16 (2**–**213)	14 ± 9 (2**–**188)	< 0.01

*COVID-19* coronavirus disease 2019, *CRP* c-reactive protein, *CT* computed tomography, *ICU* intensive care unit, *IQR* interquartile range, *LDH* lactate dehydrogenase

At baseline elderly patients exhibited severe clinical course (121/241, 50.21% vs. 98/249, 39.36%; *p* = 0.02), while non-elderly patients usually displayed a moderate-to-severe clinical course (66/241, 27.39% vs. 100/249, 40.16%; *p* < 0.01). A total of 94.08% (461/490) of the cohort necessitated oxygen supplementation upon admission to the hospital. However, a higher percentage of non-elderly patients required a low-flow nasal cannula compared to the elderly (52/225, 23.11% vs. 82/236, 34.75%; *p* < 0.01), who required a Venturi mask or non-invasive ventilation upon admission (167/225, 74.22% vs. 153/236, 64.83%; *p* = 0.03). COVID-19 infiltration ratios on chest CT scans also differed between subgroups (50 ± 37.50% vs. 50 ± 40%; *p* = 0.03). Among baseline laboratory parameters, serum CRP (140 ± 121 mg/l vs. 134 ± 121 mg/l; *p* < 0.01) and interleukin-6 levels (60.10 ± 96.73 pg/ml vs. 46.50 ± 56.00 pg/ml; *p* < 0.01) were significantly higher, while absolute lymphocyte count (0.82 ± 0.51 × 10^9^/l vs. 0.95 ± 0.59 × 10^9^/l; *p* = 0.03) and LDH (749 ± 356 U/l vs. 845 ± 443 U/l; *p* < 0.01) were significantly lower among the elderly. Serum ferritin (1020 ± 1243 ng/ml vs. 1103 ± 1137 ng/ml; *p* = 0.77) and plasma D-dimer (1276 ± 1296 ng/ml vs. 93 ± 755 ng/ml; *p* = 0.99) levels did not differ between subgroups. Remdesivir was initiated upon admission for 97.14% (476/490) of patients, and all patients received dexamethasone at baseline.

### Disease course of COVID-19

COVID-19 disease course and treatment characteristics are provided in Table 1. Among elderly patients, the time from symptom onset to hospital admission was significantly shorter (7 ± 5 days vs. 8 ± 4 days; *p* = 0.02). However, the length of hospital stay was longer (18 ± 16 days vs. 14 ± 9 days; *p* < 0.01). There were no significant differences in the timing of baricitinib therapy from COVID-19 diagnosis (2 ± 7 days vs. 4 ± 6 days; *p* = 0.07) or the occurrence of the first infectious complications from admission (19 ± 22.25 days vs. 15 ± 79 days; *p* = 0.46) or the occurrence of the first infectious complications from the initiation of baricitinib treatment (15 ± 22 days vs. 13 ± 80 days; *p* = 0.32) within either subgroup. Baricitinib was concurrently administered with tocilizumab in 9.59% of the patients (*n* = 47). Patients who received both baricitinib and tocilizumab (*n* = 47) had a comparable 1-year mortality rate (13/47, 27.66% vs. 127/443, 28.67%; *p* = 1.00), severe secondary infection rate (10/47, 21.28 vs. 87/443, 19.64; *p* = 0.85), and a non-severe secondary infection rate (4/47, 8.51 vs. 49/443, 11.06%; *p* = 0.81) with the patients receiving only one immunomodulatory treatment (*n* = 443). The requirement for intensive care unit admission was also statistically similar (85/241, 35.26% vs. 78/249, 31.33%; *p* = 0.39). Among the included patients, there were no cases in which baricitinib treatment completion became necessary due to a serious adverse event.

### Clinical and microbiological outcomes

**Fig. 2:**
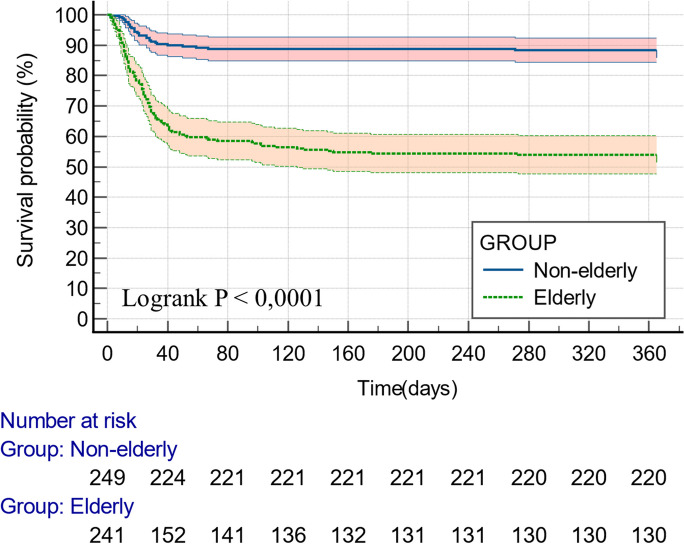
Kaplan–Meier survival curves during the 1-year follow-up period among elderly and non-elderly patients treated with baricitinib for COVID-19

Clinical and microbiological outcome data are provided in Table 2, 3, and 4. Mortality rates were notably higher among the elderly during all follow-up endpoints (30-day, 79/241, 32.78% vs. 22/249, 8.84%, *p* < 0.01; 90-day, 100/241, 41.49% vs. 28/249, 11.24%, *p* < 0.01; 1-year, 111/241, 46.06% vs. 29/249, 11.65%, *p* < 0.01). Kaplan–Meier survival curves are shown in Fig. 2. At 90 days and 1 year, severe secondary infections were more prevalent in the elderly subgroup (90 days, 56/241, 23.24% vs. 37/249, 14.86%, *p* = 0.02; 1 year, 58/241, 24.07% vs. 39/249, 15.66%, *p* = 0.02). Also, severe secondary infection incidence rate was notably higher among elderly patients during follow-up (30 days, 1.69, 1.08–2.67, *p* = 0.02; 90 days, 2.07, 1.34–3.22, *p* < 0.01; 1 year, 2.27, 1.49–3.50, *p* < 0.01). Results are provided in Table 3. Regarding different types of secondary infections, statistically significant differences were not documented between the two subgroups (Table 4.)

**Table 2:** Clinical outcomes of elderly and non-elderly patients treated with baricitinib for COVID-19 cumulated at 30-days, 90-days, and 1-year post-treatment initiation

Parameter	Total (*n* = 490)	Elderly (*n* = 241)	Non-elderly (*n* = 249)	*p*-value
At 30-day follow-up period (*n*, %)				
Death	101 (20.61)	79 (32.78)	22 (8.84)	< 0.01
Severe infection	87 (17.76)	51 (21.16)	36 (14.46)	0.06
Non-severe infection	17 (3.47)	12 (4.98)	5 (2.00)	0.09
At 90-day follow-up period (*n*, %)				
Death	128 (26.12)	100 (41.49)	28 (11.24)	< 0.01
Severe infection	93 (18.98)	56 (23.24)	37 (14.86)	0.02
Non-severe infection	27 (5.51)	13 (5.39)	14 (5.62)	1.00
At 1-year follow-up period (*n*, %)				
Death	140 (28.57)	111 (46.06)	29 (11.65)	< 0.01
Severe infection	97 (19.80)	58 (24.07)	39 (15.66)	0.02
Non-severe infection	53 (10.82)	24 (9.96)	29 (11.65)	0.56

**Table 3:** Incidence rates and incidence rate ratios of severe infectious complications in elderly and non-elderly patients treated with baricitinib for COVID-19 cumulated at 30-days, 90-days, and 1-year post-treatment initiation

Parameter	Incidence rate		Incidence rate ratio between subgroups (95% CI)	*p*-value
	Elderly (*n* = 241) (case/1000 person-time)	Non-elderly (*n* = 249) (case/1000 person-time)		
Incidence at 30 days	36.71	21.71	1.69 (1.08–2.67)	0.02
Incidence at 90 days	5.39	2.60	2.07 (1.34–3.22)	< 0.01
Incidence at 1 year	1.18	0.52	2.27 (1.49–3.50)	< 0.01

**Table 4:** Types of severe and non-severe secondary infections of elderly and non-elderly patients treated with baricitinib for COVID-19 cumulated at 1-year post-treatment initiation

Parameter	Total (*n* = 490)	Elderly (*n* = 241)	Non-elderly (*n* = 249)	*p*-value
Severe secondary infections (*n*, %)				
Bloodstream infections	54 (11.02)	31 (12.86)	23 (9.24)	0.25
Community-acquired pneumonia	1 (0.20)	1 (0.41)	0 (0)	0.49
COVID-19-associated pulmonary aspergillosis	12 (2.45)	6 (2.49)	6 (2.41)	1.00
Cytomegalovirus viraemia	2 (0.41)	0 (0)	2 (0.80)	0.50
Hospital-acquired pneumonia	8 (1.63)	1 (0.41)	7 (2.81)	0.07
Human herpesvirus 6 viraemia	1 (0.20)	0 (0)	1 (0.40)	1.00
Intraabdominal infection	2 (0.41)	1 (0.41)	1 (0.40)	1.00
Skin and soft tissue infection	2 (0.41)	1 (0.41)	1 (0.40)	1.00
Urinary tract infection	3 (0.61)	3 (1.24)	0 (0)	0.12
Ventilation-associated pneumonia	55 (11.22)	31 (12.86)	24 (9.64)	0.32
Total	140 (28.57)	75 (31.11)	65 (26.10)	0.23
Non-severe secondary infections (*n*, %)				
Acute conjuctivitis	3 (0.61)	0 (0)	3 (1.20)	0.25
Acute blepharitis	3 (0.61)	1 (0.41)	2 (0.80)	1.00
Acute bronchitis	5 (1.02)	1 (0.41)	4 (1.61)	0.37
Acute gastroenteritis	6 (1.22)	2 (0.83)	4 (1.61)	0.69
Acute keratitis	1 (0.20)	0 (0)	1 (0.40)	1.00
Acute otitis externa	4 (0.82)	3 (1.24)	1 (0.40)	0.37
Acute otitis media	2 (0.41)	0 (0)	2 (0.80)	0.50
Acute parodontitis	2 (0.41)	1 (0.41)	1 (0.40)	1.00
Acute prostatitis	1 (0.20)	0 (0)	1 (0.40)	1.00
Acute rhinosinusitis	3 (0.61)	1 (0.41)	2 (0.80)	1.00
Acute tonsillopharyngitis	8 (1.63)	1 (0.41)	7 (2.81)	0.07
Acute urocystitis	3 (0.61)	3 (1.24)	0 (0)	0.12
*Clostridioides difficile* enterocolitis	15 (3.06)	10 (4.15)	5 (2.01)	0.20
Community-acquired pneumonia	1 (0.20)	1 (0.41)	0 (0)	0.49
Herpes simplex 1 gingivostomatitis	1 (0.20)	0 (0)	1 (0.40)	1.00
Skin and soft tissue infection	4 (0.82)	2 (0.83)	2 (0.80)	1.00
Total	62 (12.65)	26 (10.79)	36 (14.46)	0.28

*COVID-19* coronavirus disease 2019

## Discussion

### Present study

We conducted a single-centre comparative observational study involving 490 hospitalized elderly and non-elderly adult patients with COVID-19 infection associated with CSS, who received baricitinib in addition to the standard-of-care treatment. A higher prevalence of female gender, essential hypertension, chronic cardiovascular disease, chronic renal disease, chronic cerebrovascular disease, diabetes mellitus, and active oncological malignancy was observed among elderly patients. A substantial 76.33% of the total cohort had not received vaccination at the time of inclusion. At baseline, elderly patients experienced more severe symptoms and required oxygen supportation with Venturi masks or non-invasive ventilation, with extended infiltrations on chest CT scans. Furthermore, a significantly lower absolute lymphocyte count, and elevated levels of interleukin-6 and CRP were recorded among this group. Regarding the time from hospitalization to baricitinib initiation and ICU admission rates, we did not detect any differences between subgroups. However, a longer hospitalization period was observed among the elderly. Furthermore, we observed a 19.80% severe secondary infection rate and a 10.82% non-severe secondary infection rate in the total cohort during the 1-year follow-up period. During the 90-day and 1-year follow-up, severe secondary infections were significantly more prevalent among elderly patients. The 30-day, 90-day, and 1-year all-cause mortality rates were also significantly higher among the elderly patients compared to the non-elderly. In conclusion, elderly patients demonstrated a propensity for long-term morbidity and mortality, irrespective of baricitinib treatment.

### Previous studies from the literature

Age and specific comorbidities are well-established risk factors for COVID-19 disease progression [8]. Differences in morbidity and mortality between elderly and non-elderly COVID-19-infected patients arise from a complex interplay of age-related mechanisms [13]. First, the pathological alteration of the immune system during aging, defined as immunosenescence, affects both innate and adaptive immunity, which contribute to chronic illnesses and a reduced capacity to effectively combat infections [14, 15]. An important aspect of immunosenescence is “inflamm-aging”, characterized by the accumulation of proinflammatory cytokines due to damaged macromolecules or cells, resulting in a low-level sterile chronic inflammation [15]. Immune cells adapt to continuous signalling, leading to a decline in their function with age. Aging also influences haematopoietic stem cells, causing a shift towards myeloid cell production, resulting in lymphopenia, a well-established prognostic factor for poorer COVID-19 outcomes [14]. With increased myeloid cell production, their function and activation rate are enhanced, altering the proinflammatory cytokine profile [15]. Upregulation of interleukin-6 and downregulation of interferon-γ further also inhibit the cellular lymphocyte response [15]. In our study, lymphopenia was significantly higher among elderly patients, reflecting the suggested cellular and subcellular mechanisms in a real-world cohort.

Furthermore, in the course of aging, there is an augmentation of cellular senescence and the senescence-associated secretory phenotype [16]. The latter compromises cytokine production by senescent cells, thereby inducing extracellular matrix degradation, a pro-inflammatory state, pro-coagulation, and an augmented complement activation [16]. The secretory phenotype disseminates in a paracrine manner among cells, thereby hastening the aforementioned processes [16]. In addition to the aging processes, it is noteworthy that SARS-CoV-2 has the capacity to induce senescence and the proinflammatory phenotype [17]. In the geriatric population, both pre-existing and virus-induced senescence concomitantly contribute to and exacerbate proinflammatory cytokine production. This, in turn, contributes to the manifestation of cytokine storm syndrome, thrombosis, and tissue injury-induced fibrosis. Consequently, this cascade leads to multiple organ failure and an elevated mortality rate among the elderly [17].

Current literature also emphasizes the role of gastrointestinal tract dysbiosis in increased morbidity and mortality among the elderly [18]. The aging process of the gastrointestinal tract is characterized by alterations in the intestinal microbiota, with an abundance of Bacteroidetes and a decrease in anti-inflammatory elements, contributing to a proinflammatory environment similar to “inflamm-aging” and a weakened intestinal barrier [19]. Additionally, microbiome studies also suggest a SARS-CoV-2-induced dysbiosis and a colonization of opportunistic pathogens in the gastrointestinal tract, facilitating secondary infections [18].

Besides the cellular and molecular mechanisms, frailty is also an age-related condition marked by bedridden states, dietary deficiencies, and polypharmacy, and is associated with higher rates of hospitalization and mortality, regardless of COVID-19 [13, 20]. At the physiological level, it can be defined as a decline in organ function and reserves, which, at a systemic level, leads to a reduction in daily activities. Beyond the age of 65, frailty affects approximately 15% of the elderly population, with a higher prevalence among female patients [13]. Geriatric hospitals, nursing homes, and long-term care facilities where frail elderly patients accumulate have been recognized as epidemiological hotspots for COVID-19 transmission, exposing them to a higher overall risk of contracting the disease [21].

Among the elderly, decreased drug bioavailability, lower levels of transport proteins due to malnutrition, hypoperfusion in peripheral blood, alterations in body fat and water balance, drug-drug interactions, and decreased liver or kidney function are expected regardless of SARS-CoV-2 infection [22]. Additionally, baricitinib is indicated for the treatment of COVID-19 infection with a severe clinical course and an associated cytokine storm [23]. This comprises an uncontrolled and hyperresponsive systemic inflammatory milieu, with an excess of proinflammatory cytokines resulting in multiorgan failure through vascular and immune-mediated pathways [23]. Therefore, baricitinib pharmacokinetics modulate both due to age-related and immune-mediated processes [22, 23]. Dose modification or the termination of the drug is indicated in cases of severely decreased kidney function (eGFR < 15 ml/min/1.73m^2^), suspicion of drug-induced liver injury (aspartate aminotransferase or alanine aminotransferase levels at least five times above the ULN, with a suspected drug-induced liver injury), severe lymphopenia (absolute lymphocyte count < 200 cells/µl), and severe neutropenia (absolute neutrophil count < 500 cells/µl) [3]. However, in our study, there were no cases that required baricitinib dose modification or necessitated drug termination due to a severe adverse event.

Previous studies in the literature have suggested that infected and hospitalized elderly patients experience significantly higher rates of secondary infections. In a single-centre observational cohort study that assessed pulmonary secondary infection rates and clinical outcomes among older adults testing positive for SARS-CoV-2, approximately 43% were diagnosed with suspected superimposed infections through positive bacterial cultures or radiological findings. The study reported increased mortality rates and extended hospitalizations specifically among elderly patients with secondary infections [9]. In comparison to our study, this report documented higher secondary infection rates, potentially attributable to the fact that included patients were likely to be bedridden, heightening the risk of pulmonary infections. An Indian retrospective observational study focused on the mortality rates of elderly patients hospitalized for COVID-19. Their findings indicated notably higher mortality rates among patients with at least three comorbidities, severe COVID-19 disease courses, and those experiencing multi-organ failure, elevated creatinine, or acute liver injury complications [24]. Similarly, a case–control observational study including 179 elderly patients hospitalized for non-COVID-19-related diseases found that 89.9% of patients contracted the disease in geriatric hospital settings. The study identified a mortality rate of 14.4% among elderly patients with a negative SARS-CoV-2 RT-PCR test, and a 29.2% mortality rate among COVID-19 patients [25]. Similar mortality rates were observed during our 1-year follow-up period among elderly patients. Furthermore, a retrospective observational study compared previously SARS-CoV-2-infected and hospitalized elderly patients with historical controls suffering from seasonal influenza between March 2020 and August 2022. The study reported a heightened long-term risk of hospital readmission or all-cause death at 30 days, 90 days, and 180 days post-discharge. The authors also noted a significant decline in all-cause mortality over the study period during the pandemic [26]. At the 1-year follow-up, we were also able to observe higher rates of hospital readmission due to severe secondary infections and mortality among the elderly.

Baricitinib received its initial approval in November 2020, demonstrating its superior efficacy in combination with remdesivir over remdesivir monotherapy among hospitalized patients receiving high-flow oxygen or non-invasive ventilation [27, 28]. Historically, in rheumatoid arthritis therapy with 2 or 4 mg baricitinib doses, there were elevated incidences of mild to moderate upper respiratory tract infections, urinary tract infections, gastroenteritis, and reactivation of herpes simplex virus [29]. Additionally, severe infectious complications, such as herpes zoster or cellulitis, were reported [29]. Subsequently, early international clinical data indicated heightened rates of secondary infections also among hospitalized SARS-CoV-2-infected patients undergoing baricitinib therapy [3, 4]. Consequently, the Hungarian National Guideline recommended prophylactic antimicrobial measures upon completing baricitinib therapy [5]. However, novel real-world clinical data did not suggest significantly elevated secondary infection rates [6, 7, 28]. A systematic review from China assessed the safety and efficacy of baricitinib in hospitalized SARS-CoV-2-infected patients, comparing outcomes with those receiving a placebo or alternative treatments. This investigation revealed reduced rates of secondary infections in the baricitinib treatment arm [28]. Moreover, a retrospective cohort study compared secondary infection rates and 28-day mortality rates among patients receiving baricitinib plus remdesivir and dexamethasone (*n* = 43) and patients receiving only remdesivir and dexamethasone (*n* = 53) [7]. Baricitinib therapy was 2 or 4 mg depending on the decision of the physicians for a maximum of 14 days [7]. According to the study result, there were no significant differences among the two study arms in the secondary infection rate or 28-day mortality rate [7]. Also, a retrospective cohort study among transplant patients (89% solid organ recipients) compared secondary infection rates between baricitinib plus SOC-treated (*n* = 77) and only SOC-treated (*n* = 114) patients [6]. Similarly, secondary infection rates did not differ among the two subgroups [6]. Fundamentally, the aforementioned evidence suggests that the variance in secondary infection rates between patients treated for rheumatoid arthritis or severe COVID-19 infection may be attributed to the duration of treatment.

Previous studies have also investigated the impact of the immunomodulatory baricitinib in COVID-19-associated CSS among the elderly. A retrospective cohort study using propensity score matching evaluated elderly patients, defined as 70 years or older, and non-elderly patients with moderate-to-severe COVID-19 pneumonitis who received baricitinib compared to those who did not. The study observed an overall 48% reduction in all-cause mortality rates and an 18.5% absolute mortality risk reduction at 30 days among elderly patients who received immunomodulatory treatment [30]. Our study differs from this research in terms of study design. Our definitions are based on different age thresholds, and we compared elderly populations to non-elderly ones, both of which received baricitinib. In contrast, the present study involved a control population that did not receive immunomodulation beyond SOC.

In conclusion, our findings align with current literature data suggesting that elderly patients experience more severe microbiological and clinical outcomes, regardless of immunomodulatory therapy.

### Limitations

Our study had limitations. Firstly, the evolution of general knowledge influenced by emerging COVID-19 evidence led to adjustments in treatment protocols. Despite dedication, occasional delays in protocol updates might have occurred due to treatment availability issues. Secondly, our retrospective study included a long patient inclusion period, so multiple SARS-CoV-2 variants were included in the study, and routine variant typing is not available at our centre. Moreover, comorbidities were more frequent among elderly patients. Age and certain comorbidities are both risk factors for COVID-19 disease progression; thus, higher mortality rates were inherently expected among the elderly group [8]. Furthermore, the male gender was more common in the non-elderly subgroup, potentially influencing the insignificant results regarding the need for mechanical ventilation or admission to the intensive care unit. Finally, residual confounding might have biased our results during the 1-year follow-up period.

### Future research directions

In our study, we found that elderly patients exhibited a tendency towards enduring morbidity and mortality, independent of baricitinib treatment. Certain national guidelines, such as the one in Hungary, recommended prophylactic antimicrobial measures and suggested a 3- to 6-month regimen of co-trimoxazole and acyclovir following the completion of baricitinib [5]. For future research, a redefinition of prophylactic requirements among both elderly and non-elderly patient subpopulations should probably be an intriguing question.

## Conclusion

According to our findings, elderly patients with severe SARS-CoV-2 infection and cytokine storm syndrome (CSS) demonstrate a more severe morbidity burden with elevated secondary infection rates and increased long-term mortality, irrespective of the administration of baricitinib, during follow-up.

## Data Availability

Anonymised data of patients are available from the corresponding author upon reasonable request.

## Abbreviations

ACE-2: Angiotensin-converting enzyme 2
CI: Confidence interval
COVID-19: Coronavirus disease 2019
CRP: C-reactive protein
CSS: Cytokine storm syndrome
CT: Computed tomography
DNA: Deoxyribonucleic acid
eGFR: Estimated glomerular filtration rate
ESCMID: European Society of Clinical Microbiology and Infectious Diseases
ICU: Intensive care unit
ICU LOS: Intensive care unit length-of-stay
IDSA: Infectious Diseases Society of America
IQR: Interquartile of range
LDH: Lactate dehydrogenase
LOS: Length-of-stay
RT-PCR: Real-time polymerase chain reaction
SARS-CoV-2: Severe acute respiratory syndrome coronavirus 2
SOC: Standard-of-care
ULN: Upper limit of normal
WHO: World Health Organization
